# Environmental Determinants of Health

**DOI:** 10.1093/geroni/igaf122.245

**Published:** 2025-12-31

## Abstract

In this session research will be presented on the impact to the environment on healthy aging

